# Quantifying the effect of media limitations on outbreak data in a global online web-crawling epidemic intelligence system, 2008–2011

**DOI:** 10.3402/ehtj.v6i0.21621

**Published:** 2013-11-08

**Authors:** David Scales, Alexei Zelenev, John S. Brownstein

**Affiliations:** 1 Children's Hospital Informatics Program, Boston Children's Hospital, Harvard Medical School, Boston, MA, USA; 2 Center for Biomedical Informatics, Boston Children's Hospital, Harvard University, Boston, MA, USA; 3 Department of Internal Medicine, Section of Infectious Disease, Yale University School of Medicine, New Haven, CT, USA

**Keywords:** epidemic intelligence, infectious diseases, system evaluation, HealthMap, crowd out effect

## Abstract

**Background:**

This is the first study quantitatively evaluating the effect that media-related limitations have on data from an automated epidemic intelligence system.

**Methods:**

We modeled time series of HealthMap's two main data feeds, Google News and Moreover, to test for evidence of two potential limitations: first, human resources constraints, and second, high-profile outbreaks “crowding out” coverage of other infectious diseases.

**Results:**

Google News events declined by 58.3%, 65.9%, and 14.7% on Saturday, Sunday and Monday, respectively, relative to other weekdays. Events were reduced by 27.4% during Christmas/New Years weeks and 33.6% lower during American Thanksgiving week than during an average week for Google News. Moreover data yielded similar results with the addition of Memorial Day (US) being associated with a 36.2% reduction in events. Other holiday effects were not statistically significant. We found evidence for a crowd out phenomenon for influenza/H1N1, where a 50% increase in influenza events corresponded with a 4% decline in other disease events for Google News only. Other prominent diseases in this database – avian influenza (H5N1), cholera, or foodborne illness – were not associated with a crowd out phenomenon.

**Conclusions:**

These results provide quantitative evidence for the limited impact of editorial biases on HealthMap's web-crawling epidemic intelligence.

In the past decade, news-based epidemic intelligence services engaged in early outbreak detection have seen growth in both number and prominence (1, 2). Organizations such as the World Health Organization (WHO) often initially discover outbreak events via informal sources (3–5), such as Global Public Health Information Network (GPHIN) (6), BioCaster (7), Pattern-based Understanding and Learning System (PULS) (8), EpiSPIDER (9), MedISys (8) and HealthMap (10). While the utility of such web-crawling systems has been well established (2, 7, 11–13), there are no publicly available evaluations quantifying the limits of news-based epidemic intelligence based on external factors and only one study descriptively examines potential limitations (14). The aim of this article is to categorize and quantify certain constraints from news media that may be transmitted to the epidemic intelligence data found in these systems. Such analysis will help to identify when informal intelligence is less likely to be influenced by potential bias or when significant gaps in epidemic data likely exist that may necessitate supplementation with other sources.

## Media limitations

The web-crawling epidemic intelligence systems mentioned above all have similar reliance on news articles to generate infectious disease event data. Consequently, any persistent constraints or biases in news media have the potential to affect aggregate data at either national or global scales. A full review of media bias in health coverage is beyond the scope of this article. However, in this study, we test for and quantify the effect of two key media limitations that are likely to be replicated in epidemic intelligence using news-based sources: human resource constraints and an editorial bias.

Human resource constraints are an inevitable consequence of the fact that media production is a labor-intensive endeavor. After the 2008 financial crisis, during which there was a precipitous drop in advertising revenue and news budgets in many countries, particularly in print sources, human resource constraints are likely to be even more acute (15). As a result, we might expect a decline in event-based reporting via news media during times when news staff have declined relative to baseline, such as on weekends or during certain holidays. While these human resource constraints are widely recognized in the often hyperbolized reporting about the ‘death of newspapers’ (16, 17), a quantitative link between these constraints and content-specific declines in media coverage has yet to be established. Only one study implies a link between declines in reporters covering state capitals in each of the 50 US states and reduced political coverage but the link is not verified quantitatively (18). We hypothesize that news-based epidemiological monitoring systems would see a decline in the number of reported events coinciding with weekends and major US and European holidays.

Media coverage of health stories also suffers from an editorial bias. Studies of health in the media have primarily focused on the portrayal of risk (19), disasters, war and death (20). These studies describe biases that are also likely to appear in reporting on epidemic disease, namely the economic drive to sell newspapers and/or advertisements fosters ‘if it bleeds it leads’ sensationalism (21) and focuses on shocking stories and casualty numbers (22). Not all outbreaks are treated equally, however, as western media coverage of humanitarian disasters is not always correlated with disaster severity (23, 24). We hypothesize that a sensationalistic editorial bias in news media can lead to a ‘crowd out’ phenomenon in which large numbers of reports on certain diseases and epidemics can temporarily crowd out reportage of other, less sensational, disease events. In our study, we examine whether media attention may be pre-empted by competing disease events by quantifying any declines in other disease coverage.

## Methods

### Data collection and database

English-language data deemed a ‘breaking’ alert from June 1, 2008 to May 31, 2011 was taken from HealthMap's database of emerging infectious diseases events (for more on HealthMap, see Ref. 10). Data were limited to that which had been precisely located by HealthMap's system of curators, all of which have a public health background. Events are labeled ‘breaking’ if they contain information about a new outbreak or new information about ongoing outbreaks (increased case counts, further geographic spread, etc.).

During this time period, HealthMap's data are derived from the media aggregators Google News and Moreover. According to their respective websites, these aggregators do not list what sources are included in their feeds. Therefore, the sources provided by them potentially change in both quantity and content over time. While both feeds claim global coverage, preliminary analysis of media sources shows that US domestic news sources predominate in Google News while Moreover contributes more international news stories to the database (data not shown). Due to these geographic discrepancies, the two feeds were analyzed separately.

**Fig. 1:**
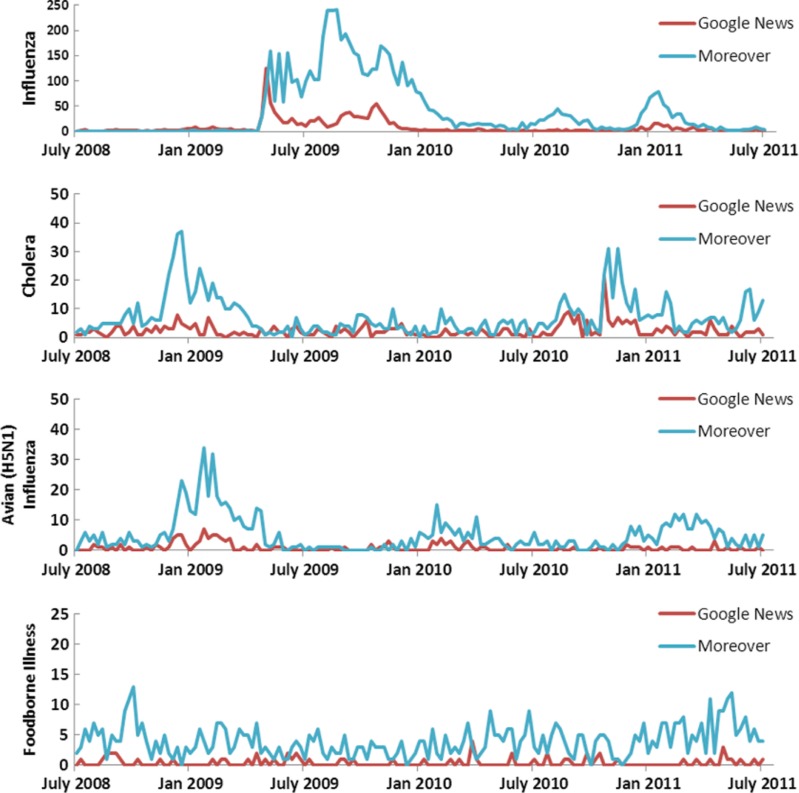
Time series by disease and feed for top diseases by number of events in the HealthMap database. *Note*: influenza includes both untyped influenza and ‘swine flu’ H1N1 events. Number of HealthMap Alerts by Disease, July 2008–July 2011.

A full list of diseases can be found on HealthMap's website: www.healthmap.org. Diseases used to test for editorial bias were selected by the total number of times they appeared in the HealthMap database. The top two diseases are influenza and ‘swine flu’ (H1N1), which are listed separately in the database, followed by avian influenza (H5N1), foodborne illness and cholera (Fig. 1).

### Statistical analysis

The principal method used to test for the aforementioned media biases involves modeling the time series for total disease outbreak events. As the hypothesized media biases have two different causes, our analysis will consist of two different approaches. First, the human resource constraint is influenced by deterministic events and merits analysis of the frequency domain via variables that measure periodicity. Second, the expected crowd out phenomenon is not deterministic but stochastic and might be driven by other concurrent epidemics. Therefore, we built a stochastic model to identify and extract the signal in the aggregate data due to co-movements with other outbreaks.

#### Part I: Indirect test for human resource constraints

Here, we examine the data's cyclical pattern to isolate and identify the effect of potential human resource constraints. To achieve this goal, we estimate a series of ordinary least square (OLS) regressions. The first specification examines alert count fluctuations based on days of the week. Our dependent variable is the number total outbreak events per day; the independent variables are dummy variables indicating weekdays (i.e. Monday, Tuesday, Wednesday, and so on), and the model is estimated at a daily frequency. The second specification examines alert count fluctuations that may correspond with particular holidays. Here, we estimate the model at a weekly frequency where the dependent variable is the number of total outbreak events per week and the independent variables are dummy variables for each of the weeks when a specific US and European holiday occurred. The daily data are aggregated to produce a weekly series in order to smooth out the daily variation. For the holiday analysis, we ran univariate models first, and then used an adjusted *R* ^2^ to evaluate relative goodness of fit and select our multivariate model. Holidays selected were major federal holidays in the United States and major religious and secular holidays common across European countries, including the two largest Muslim holidays. We excluded international holidays that were unlikely to have a large impact on media coverage in our English-language dataset (e.g., Chinese New Year).

#### Part II: Assessing the magnitude of the crowd out effect

To identify potential editorial bias (i.e. crowd out effect), we estimate the amount that disease events of different types displace one another using an auto-regressive integrated moving average (ARIMA) model and the Box-Jenkins approach (25, 26). This analytical approach differs from the previous analysis because the crowd out phenomenon is stochastic and contingent on the concurrence of other disease outbreaks. Unlike most regression techniques including the Poisson regression, time series methods account for correlation structure in the data, presence of trends, unit roots and various cyclical fluctuations. Failure to account properly for time series structures of the data will result in a misspecified model which may lead to a biased estimation and inaccurate inference (27). ARIMA models (25) were designed to take the time structure of the data into account, and for this reason have been widely used in detecting outbreaks of infectious disease (28–31).

In ARIMA models, the dependent variable is thought to consist of two main components: an auto-regressive part and a moving-average part. The former is modeled based on past values of the dependent variable, as well as current and past values of independent variables, which are observed. The latter is modeled as a summation of error terms that exhibit correlation across time.

To properly identify the structure of the ARIMA model, we adopt Box-Jenkins four-step analytical procedure, which we use to test the data for time series properties (26). The first two steps examine various time series properties of the data to identify the proper model, the third step estimates the model, and the fourth step validates it.

First, we analyzed the mean-range plots to determine whether the variance of the series needed to be stabilized, which in our case required a logarithmic transformation. We also assessed the stationarity of each series, a property which implies that the distribution of the variable, including mean and variance, does not depend on time. If any of the variables in a regression framework are not stationary and contain unit roots, the standard assumption of the asymptotic variance may not be valid (26). As a result, the usual *t*-ratios used in the hypothesis testing of the parameters may not follow a *t*-distribution. To test for stationarity of the series, we performed the Phillips–Perron (PP) test and the Augmented Dickey–Fuller (ADF) test that a variable has a unit root; the alternative hypothesis is that the variable was generated by a stationary process (32, 33).

Second, we combined graphical and analytical methods to determine the specification of the model based on the best time series representation of each series. Specifically, we estimated auto-correlation and partial auto-correlation functions and examined their plots to identify the proper auto-regressive and moving-average components. As we were interested in measuring the crowd out effect by incorporating other disease variables as covariates, we analyzed cross-correlation functions between total reported outbreaks and each of the four disease series. It is very likely that the auto-correlation properties of each series could influence cross-correlations and lead to incorrect conclusions about temporal relationships between variables (29). To avoid this, we employed a ‘pre-whitening’ technique in which we first applied an ARIMA model to each of the four series, we then fit an ARIMA model for the aggregate disease series, and we cross-correlated the residuals from the aggregate series with the residuals from each of the individual series. The cross-correlation allows us to identify the proper temporal order of the covariates, since cross-correlations capture the degree of co-movement between data series across time. Knowing the proper temporal order of the variables can help us identify the statistical model that will measure the crowd out effect, since the number of total outbreak events is likely to depend on its own history and on the timing and history of other outbreak events.

Third, we estimated the ARIMA models using OLS regression with Newey–West standard errors (34). In the case where the integrated order was 1 and the series was non-stationary (e.g., influenza for the Moreover series) we included an additional lag of the series to make sure the error term would become stationary (35, 36). We selected a model that fit our data best and was relatively parsimonious based on Akaike Information Criterion (AIC). Finally, we employed Ljung-Box test to verify that the residuals constituted white noise and the assumptions of the model were satisfied. STATA v12 was used for the data analysis (37).

## Results

**Fig. 2:**
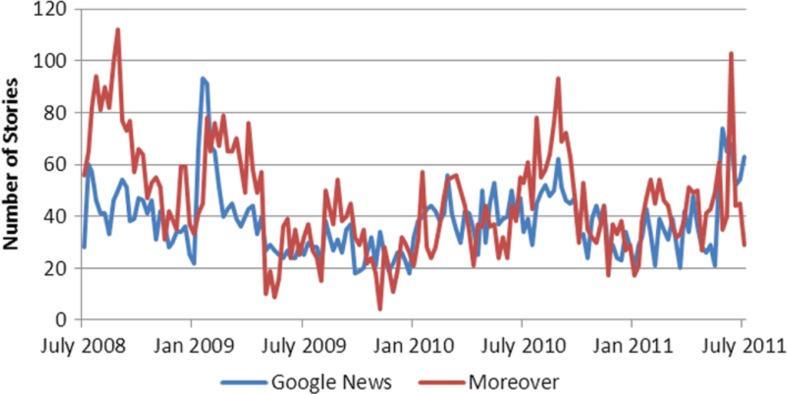
Breaking disease^1^ alerts by week over the study period. 1. The following diseases are excluded from this series: influeza influenza/H1N1, cholera, avian influenza and foodborne illness.

Plotting observed total alerts (Fig. 2) from Google News and Moreover series reveals a similar pattern of fluctuations. Both the PP as well as the ADF tests confirm that both of the aggregate series are covariance stationary, as evidenced by highly significant test statistics (data not shown), implying that the series revert back to each of their long-run averages.

The Box-Jenkins methodology provides evidence that Google News and Moreover series are best described by auto-regressive processes of first and second order, respectively, based on the auto-correlation functions, the AIC and the Ljung-Box test (data not shown). This implies that the best predictor of the current number of disease events in Google News is yesterday's number of disease events, while the best predictor of current number of disease event in Moreover is both yesterday's and the previous day's disease events.

Taken together, the stationarity tests and analysis of auto-correlation functions imply that the OLS method for estimation of the crowd out effect and human resource constraint biases in the series are appropriate. The conclusion is also confirmed when we validate our OLS model by the Ljung-Box test, which confirms that errors behave as ‘white noise’ and satisfy the assumptions of the OLS models.

### Human resource constraints

**Fig. 3:**
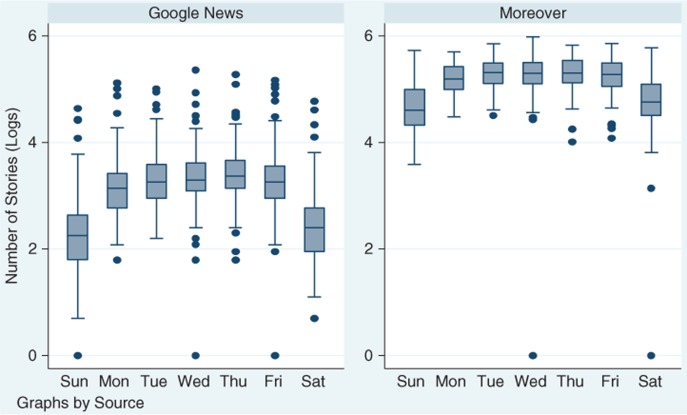
Natural log of the number of HealthMap alerts by day of the week. *Note*: the rectangle marks 25th and 75th percentile distributions; the middle line is the median; whiskers mark the upper adjacent line (which represents 1.5 times the interquartile range); the dots represent extreme values and outliers.

The analysis for Part 1 provides evidence for human resource constraints (Tables 1 and 2). Because the models are estimated as semi-log equations, the coefficients can be converted into percent changes in the outcome according to formulas from Halvorsen and Palmquist (38, 39). We find that the total number of reported outbreak events declines dramatically during the weekend (Fig. 3). For Google News, the number of alerts declined by 58.3% on Saturday and 65.9% on Sunday relative to Tuesday, Wednesday and Thursday. A similar pattern was observed in Moreover data where the number of alerts dropped by 39.8% on Saturday and 46.6% Sunday relative to Tuesday, Wednesday and Thursday. The number of alerts on Monday is on average 14.7% lower than the number of events reported on Tuesday in Google News and 9.2% lower relative to Tuesday, Wednesday and Thursday in Moreover.

**Table 1:** The influence of day of the week on log of total disease alerts*

	Google News			Moreover		
		
Weekday (*N*=1,097)	% Change**	95% CI**	*p*	% Change**	95% CI**	*p*
Monday	**−14.7**	**(−20.3, −9.1))**	**0.02**	**−9.2**	**(−14.6, −7.2)**	**0.04**
Tuesday	Referent			Referent		
Wednesday	1.4	(−3.1, 6.0)	0.84	−2.8	(−7.6, 1.1)	0.53
Thursday	7.8	(−3.7, 19.2)	0.26	1.5	(−6.7, 7.3)	0.75
Friday	−3.3	(−16.9, 10.3)	0.67	−3.2	**(−11.9, 4.6)**	0.48
Saturday	**−58.3**	**(−72.7, −43.8)**	**<0.01**	**−39.8**	**(−48.9, −30.0)**	**<0.01**
Sunday	**−65.9**	**(−78.9, −52.9)**	**<0.01**	**−46.6**	**(−55.3, −34.8)**	**<0.01**
Adjusted *R* ^2^			0.34			0.27

* Including influenza/H1N1, cholera, avian influenza and foodborne illness.

** Calculation based on formula from Halvorsen and Palmquist (38) applied to OLS coefficient; The Delta method (formula for which can be found in Greene (39)), was used in the computation of the confidence intervals.

OLS, ordinary least square.

Bold values indicate instances where *p*<0.05.

**Table 2:** The influence of holidays on log of total disease-related events by source (2008–2011)*

	Google News						Moreover					
		
	Univariate			Multivariate			Univariate			Multivariate		
				
International holidays (*N*=157)	% Change	95% CI	*p*	% Change	95% CI	*p*	% Change	95% CI	*p*	% Change	95% CI	*p*
International holidays (religious)												
Christmas and New Years'	**−27.4**	**(−41.3, −12.6)**	**<0.01**	**−27.4**	**(−41.4, −12.7)**	**<0.01**	−19.7	(−38.7, −0.1)	0.08	−20.1	(−39.5, −0.6)	0.07
Easter	3.0	(−21.1, 26.5)	0.8				−7.7	(−42.3, 26.3)	0.66			
Eid Al Adha	−15.6	(−49.6, 18.5)	0.4				−23.7	(−53.0, 5.8)	0.17			
Eid Al Fitr	16.2	(−3.0, 36.3)	0.1				**37.7**	**(8.7, 68.0)**	**<0.01**	17.7	(−11.0, 46.5)	0.19
European holidays												
Labor day (Europe)	−13.1	(−31.2, 5.5)	0.2				15.0	(−5.6, 35.8)	0.13			
Europe day (May 9)	−9.5	(−33.0, 14.7)	0.5				−39.3	(−84.5, 5.8)	0.19			
Armistice day	5.1	(−6.6, 16.9)	0.4				−52.8	(−113.4, 8.52)	0.26			
US holidays												
Martin Luther King day	37.7	(−34.8, 109.5)	0.2				12.7	(−10.2, 36.2)	0.25			
Presidents day	13.9	(−8.4, 35.2)	0.2				19.7	(−15.4, 53.8)	0.24			
Memorial day (US)	16.2	(−43.2, 74.9)	0.6				**−36.2**	**(−62.2, −10.6)**	**0.03**	**−37.0**	**(−62.9, −10.9)**	**0.03**
Independence day (July 4)	10.5	(−32.2, 52.4)	0.6				−17.3	(−48.8, 13.5)	0.32			
Labor day (US)	12.7	(−27.9, 53.2)	0.5				64.9	(−19.3, 148.1)	0.06	51.7	(−36.0, 139.3)	0.16
Columbus day	−13.9	(−47.6, 20.3)	0.5				20.9	(−15.1, 56.8)	0.22			
Veteran's day (US)	5.1	(−6.56, 16.9)	0.4				−52.8	(−113.3, 8.53)	0.26			
Thanksgiving (US)	**−33.6**	**(−47.0, −20.7)**	**<0.01**	**−34.9**	**(−47.7, −21.7)**	**<0.01**	**−41.1**	**(−67.9, −14.3)**	**0.02**	**−41.6**	**(−68.5, −14.5)**	**0.02**
Other events:												
US election (2008)	8.3	(−5.4, 22.8)	0.2				13.9	(−8.70, 37.29)	0.2			
Adjusted *R* ^2^				0.05						0.05		

* Calculation of percent change based on formula from Halvorsen and Palmquist (38) applied to OLS coefficients; Delta Method, as described in Greene (39), was used in the computation of 95% confidene interval.

OLS, ordinary least square.

Bold values indicate instances where *p*<0.05.

As previously mentioned, using a weekly series, we find evidence for a reduction in reported number of outbreaks during weeks that contain US and European holidays. In Google News, the number of reported outbreaks was 27% lower during the week of Christmas/New Year and 35% lower during Thanksgiving week than during an ‘average week’ in the year. In Moreover data, the number of reported outbreaks was 20% lower during Christmas, though not statistically significant, and 42% lower during Thanksgiving than during an average week in the year. In addition, the number of reported outbreaks was 37% lower during a Memorial Day week relative to an average week in the sample period. Other holiday effects were not statistically significant at 5% levels.

### Crowd out

Analysis of cross-correlations between total disease alerts and the set of selected diseases offers evidence for some crowd out effect (data not shown). More specifically, we found that several of the series such as influenza (Google News and Moreover), cholera (Moreover) and foodborne illness (Moreover) had lags which were negatively correlated with total disease stories.

We used this insight about cross-correlations to properly specify eight different crowd out models (four series for each of the two sources). The estimates are contained in Table 3. As all of the variables are in log units, the coefficients give us elasticities, that is, measuring a percent change in total disease stories due to a 1% change in selected disease stories.

**Table 3:** Measurement of crowd out effect based on OLS specification (with Newey–West standard errors) for Google News and Moreover

Crowd out (*N*=155) Outcome = total disease stories	Covariate	Time	Coefficient	95% CI	*p*	Adj-*R* ^2^	AIC	Ljung-Box *p*
Google	Influenza	Lag(1)	**−0.08**	**(−0.11, −0.03)**	**0.00**	0.45	−0.60	0.18
	Cholera	Contemp.	−0.03	(−0.12, 0 .06)	0.51	0.42	5.23	0.22
	Avian influenza	Lead(1)	0.07	(−0.02, 0.15)	0.09	0.40	12.20	0.15
	Foodborne illness	Contemp.	0.02	(−0.03,0.07)	0.61	0.39	15.70	0.06
Moreover	Influenza	Lag(1)	**−0.2**	**(−0.38, −0.01)**	**0.04**	0.47	124.30	0.02
		Lag(2)	0.15	(−0.04, 0.32)	0.12			
	Cholera	Lag(1)	0.02	(−0.06, 0.11)	0.67	0.46	124.30	0.27
	Avian influenza	Lag(2)	0.02	(−0.04,0.09)	0.55	0.45	124.20	0.26
	Foodborne illness	Contemp.	0.04	(−0.01,0.1)	0.48	0.46	125.00	0.21

OLS, ordinary least square.

Bold values indicate instances where *p*<0.05.

#### Influenza

The null hypothesis that the Google News influenza series is stationary cannot be rejected at 5% level. The partial auto-correlation function of the influenza series displays a sharp cutoff after the first lag, and the auto-correlation function decays more slowly, suggesting an auto-regressive pattern. Examination of the cross-correlogram reveals that the highest correlation occurs between influenza lagged once and total disease outbreak events (data not shown), implying that the association is highest between today's total disease events and yesterday's influenza events. These results are important for setting up the final model that measures the potential crowd out phenomenon. Our findings suggest that the potential for influenza to crowd out other diseases in the series is substantial: for a 1% change in influenza stories, there is a decline in 0.08% in total disease stories. Equivalently, the linear model implies that a 50% increase in influenza stories is associated with a 4% decline in total disease stories on average in the subsequent period (Table 3).

A similar pattern is present in Moreover, but due to series non-stationarity, we could not provide definitive confirmation for the presence of the crowd out effect to the same extent as in Google News. The cross-correlogram suggested that the highest correlation occurred between influenza lagged twice and total disease outbreak reports. By including two lags of the Moreover influenza variable, we are able to estimate residuals that approach white noise at 5% confidence level, which suggests that our model might fit relatively well. The estimated coefficients imply that the largest impact on total stories is observed at the start of an influenza epidemic (with a 0.2% decline in total stories for each 1% increase in influenza); as an influenza epidemic persists over multiple weeks, on average the net decline of total disease stories amounts to 0.05% per week for each increase in 1% of influenza stories per week. Even though we found the influenza parameters to be jointly significant at the 5% level, we could not confirm the validity of the *t*-test statistic and confidence intervals, as the Moreover influenza series was not stationary.

#### Cholera

The null hypothesis that Google News and Moreover cholera series were stationary could not be rejected at 5% level. Based on the analysis, the best ARIMA representation of the Google News series is a first-order, auto-regressive process, meaning that the best predictor of the current data point is yesterday's value. From the cross-correlations, we found that cholera did not have a distinct time period that exhibited the highest correlation with total disease stories. We did find that past values tended to be negatively correlated with current values of total disease stories. Examining various lag structures of cholera as predictors of total disease stories, we found that the third lag had a particularly high negative correlation with total stories; however, once we controlled for more recent lags of cholera outbreaks, we found that the relationship failed to remain significant. As a result, in our final model, we entered the contemporaneous value of cholera. The crowd out coefficient for contemporaneous cholera was slightly negative but not statistically distinct from zero. Similarly for Moreover, the best ARIMA representation for the series is a first-order, auto-regressive moving-average (ARIMA [1,0,1]) process, implying a strong time dependence in the series as well as in the error term. The highest correlation between cholera and total disease in Moreover data occurs for cholera lagged 1 week. We find no statistically significant crowd out effect from cholera on total reported disease outbreaks in the Moreover data.

#### Avian influenza (H5N1)

The analysis provides evidence that avian influenza (H5N1) is a persistent yet stationary process. The best representation of the Moreover and Google News series is a second-order, auto-regressive process, implying that the best predictors of the current number of outbreak events are the number of outbreaks from yesterday and the day before. For Google News, the highest correlation between avian influenza and total reported disease outbreaks occurs at a lead of one, implying that the number of avian flu cases reported on average increases (or decreases) one period after an increase (or decrease) in the number of reported outbreaks. We test for the co-movement of the two series directly in a regression framework, and find no statistically significant evidence for the association in the Google News data at the 5% confidence level.

In the Moreover data, the highest correlation between avian influenza and total disease outbreaks occurs when avian influenza series is lagged 2 weeks back. Again, we test the hypothesis of a crowd out effect, and find no evidence that avian influenza crowds out the total reported disease outbreaks since the parameter for crowd out is not significantly different from zero at a 5% confidence level.

#### Foodborne illness

In the Google News dataset, the reporting of foodborne illness is a high frequency series with one to two reports of the disease being the most common outcome. Examining the cross-correlograms, we found that foodborne illness was not particularly strongly correlated with total disease outbreak events for most of the lags and leads so foodborne illness variable was entered to coincide with total disease outbreaks in the final model. We found no evidence that foodborne illness crowds out total disease outbreak events in Google News since the parameter for foodborne illness variable is not significant at 5% level.

In the Moreover data, foodborne illnesses series has a higher range of values, with the largest count being 13 reported stories in 1 week. The null hypothesis that the series is stationary cannot be rejected at the 5% confidence level. The highest correlation between foodborne illness and total disease stories occurs with the contemporaneous foodborne illness values; therefore, no lag structure was specified in the final model. The statistical analysis confirms that the crowd out of foodborne illness on total disease outbreak reports is not statistically different from zero at 5% confidence level.

## Discussion

This study reveals the quantitative extent to which human resource constraints and editorial biases affect HealthMap's web-crawling epidemic intelligence. We hypothesized that human resource constraints would affect coverage of disease events during periods, where newsroom personnel might be away from their jobs. Indeed, weekends are associated with decreased numbers of events, particularly Sundays, and no suggestion of a rebound effect on Mondays. Few US holidays are associated with declines in epidemic-related news articles. Thanksgiving, Christmas/New Year (Google News) and US Memorial Day (Moreover) were the only holidays associated with significant declines in coverage. The international nature of both news feeds likely means that a more geographically-focused analysis may show more associations. However, these results imply that supplemental sources beyond informal epidemic intelligence systems may be required during weeks with common holidays in English-speaking countries.

We also tested for an editorial bias toward the most frequent diseases in HealthMap's database, finding one only for influenza in Google News, and possibly influenza in Moreover. In both cases, every 1% increase in disease-specific alerts per week was associated with a decline in other events by approximately 0.05–0.08%. When influenza events are low, the effect on overall epidemic monitoring is negligible. But during periods of high influenza activity, such as during the 2009 H1N1 ‘swine flu’ pandemic, other diseases were receiving significantly less coverage than baseline. Other diseases such as cholera, foodborne illness, avian influenza or dengue were not associated with similar crowd out effects and the Moreover feed was also not associated with a crowd out effect for any disease.

As the first study attempting to quantify the effect of media constraints and bias on web-crawling epidemic intelligence systems, we believe this research provides an important contribution to understanding the strengths and weaknesses of such informal epidemic monitoring data. However, this study is still subject to a number of limitations and weaknesses. It is worth noting that while we suggest that human resource constraints are the likely cause of the observed weekend and holiday effects, we cannot reject the possibility that there may be other as-yet unobserved sources of variability that can explain these fluctuations in HealthMap data. In particular, this study is unable to determine whether the detected decreases in infectious disease events during weekends and certain holidays are specifically due to a relative paucity of journalists covering disease stories, due to a relative lack of public health officials providing information to journalists during these time periods, or some other cause. Also, limiting our data to English biases our analysis the Anglophone world. Our global scope raises the potential for missing media bias trends at the country or local level. If our hypothesis that human resource constraints affect disease outbreak reporting is true, then country-specific analysis based on local holidays would provide for better inference and more accurate analysis. Additionally, editorial customs differ in each country, suggesting any potential crowd out effect may also depend on local context. Unfortunately, the data required for these analyses are not yet available in the HealthMap database but measures are being implemented to address this in future research. Additionally, this analysis only contains results from HealthMap, hindering the possibility of generalizing the results to other web-crawling epidemic monitoring systems.

The results of this study have practical implications for two main groups: the owners and maintainers of web-crawling epidemic intelligence systems (GPHIN, BioCaster, PULS, EpiSPIDER, MedISys and HealthMap) and the thousands using these systems daily for information about emerging epidemics. We recommend that other web-crawling systems examine their data similarly to check for these media biases and report these results to their users, particularly because there appears to be relatively little overlap in their respective data (13). HealthMap is examining the feasibility of detecting these biases in real time and posting warnings to the user when a threshold is reached. In addition, links to these results will be found on HealthMap's website to enable our users to be better informed about the data we collect. Any one web-crawling system will not always be the first to detect every new disease outbreak but each system can be transparent about their respective weaknesses to give epidemiologists greater confidence in reported results.

This study is likely to affect the practice of users of web-crawling epidemic intelligence systems for two reasons. First, a number of HealthMap users have anecdotally stated that HealthMap's reliance on unofficial information from news media sources introduces biases that render the information collected by web-crawling systems suspect. These results could assuage those fears of bias to a degree. While impacted by human resource constraints, HealthMap data, and Moreover data in particular, is resistant to an editorial bias at a global scale, perhaps because it draws from such a wide range of sources. Our users should be aware of the potential for decreased coverage during weekends and certain holidays and perhaps seek additional information from other sources during those times.

More research will need to be done to understand how potential media constraints and biases are affecting other web-crawling epidemic intelligence systems. In addition, further research should examine the impact of human resource constraints and editorial bias at the lowest geographic level possible, such as at country, province or even city level. Finally, because the results of these studies affect real-time epidemic intelligence, research should be done investigating the potential to highlight probable declines in media coverage as close to real time as possible.
